# Brugada Syndrome: The Syndrome of Right Bundle Branch Block, ST segment Elevation in V_1_ to V_3_ and Sudden Death

**Published:** 2001-10-01

**Authors:** Josep Brugada, Pedro Brugada, Ramon Brugada

**Affiliations:** 1 Arrhythmia Unit, Cardiovascular Institute, Hospital Clinic, University of Barcelona, Spain; 2 Cardiovascular Research and Teaching Institute Aalst, Cardiovascular Center, Aalst, Belgium; 3 Department of Cardiology, Baylor College of Medicine. Houston. Texas, USA

## Introduction

In 1992 a new syndrome consisting of syncopal episodes and/or sudden death in patients with a structurally normal heart and a characteristic electrocardiogram (ECG) with a pattern of right bundle branch block with an ST segment elevation in leads V1 to V3 was described [1] In 1998 the poor prognosis of patients with the syndrome not receiving an implantable defibrillator was reported [2,3] In 1998 the genetic nature of the disease and its assotiation to a mutation in the cardiac sodium channel gene was described [4]. Because the diagnosis is easily made by means of the ECG, an increasing number of patients with the ECG pattern are being identified worldwide. In this article we will review our present knowledge concerning patients with the classical ECG pattern of the disease.

**Figure 1:**
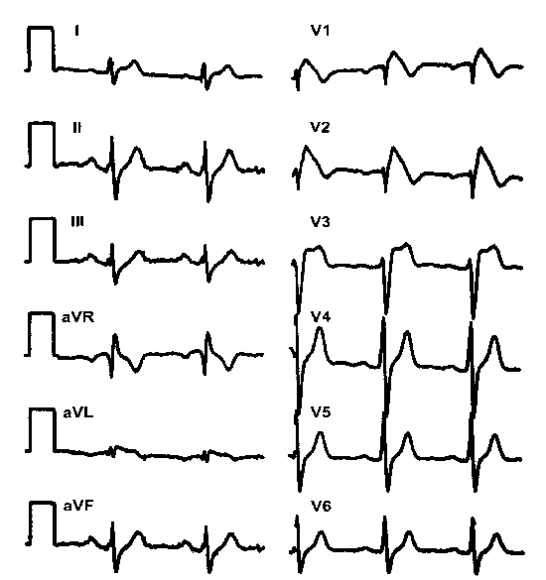
Typical ECG of the syndrome. Please note the pattern resembling a right bundle branch block in lead V1 and the ST segment elevation in leads V1 to V3. Paper speed 25 mm/s.

In the Brugada syndrome, the diagnosis is based on the history of aborted sudden death with the typical electrocardiographic pattern of ST segment elevation in leads V1-V3, with or without right bundle branch block [1] (Figure 1). In some cases, however, the diagnosis is different because some individuals present with an abnormal electrocardiogram but are completely asymptomatic or there is a history of sudden death in the family and the electrocardiographic criteria are observed.

## Incidence of an abnormal ECG in the general population

A prospective study of an adult Japanese population (22,027 subjects) showed an incidence of 0.05% of ECG's compatible with the syndrome (12 subjects) [5]. A second study of adults in Awa (Japan) showed an incidence of 0.6 % (66 cases out of 10,420) [6]. However, a third study in children from Japan showed an incidence of ECG's compatible with the syndrome of only 0.0006% (1 case in 163,110) [7]. These results suggest that the syndrome manifests primarily during adulthood, which is in concordance with the mean age of sudden death victims (35 to 40 years).

The presence of concealed and intermittent forms, however, makes the diagnosis difficult in some patients. The ECG can be modulated by changes in autonomic balance and the administration of antiarrhythmic drugs [8]. Beta-adrenergic stimulation normalises the ECG, while IV ajmaline, flecainide or procainamide accentuate the ST segment elevation and are capable of unmasking concealed and intermittent forms of the disease. These data are very important when we deal with family members of a patient with the syndrome. We know that a normal ECG in the resting state is not sufficient to exclude that a family member is affected with the syndrome. Some family members only manifest the typical ECG pattern after ajmaline or flecainide administration.

Recent data suggest that loss of the action potential dome in right ventricular epicardium but not endocardium underlies the ST segment elevation seen in the Brugada syndrome [9,1]. Also, electrical heterogeneity within right ventricular epicardium leads to the development of closely coupled extrasystoles via a phase 2 reentrant mechanism, which then precipitate ventricular tachycardia-ventricular fibrillation. Right ventricular epicardium is preferentially affected because of the predominance of transient outward current in this tissue.

## Clinical manifestations

The **complete syndrome** is characterised by episodes of rapid polymorphic VT in patients with an ECG pattern of right bundle branch block and ST segment elevation in leads V1 to V3. The manifestations of the syndrome are caused by episodes of polymorphic VT/VF. When the episodes terminate spontaneously the patient develops syncopal attacks. When the episodes are sustained, cardiac arrest and eventually sudden death occur.

There exist **asymptomatic** individuals in whom the atypical ECG is detected during routine examination. This ECG cannot be distinguished from that of symptomatic patients. In other patients, the characteristic ECG is recorded during screening after the sudden death of a family member with the disease.

On the other hand, there is the group of **symptomatic** patients who have been diagnosed as suffering syncopal episodes of unknown cause, or vaso-vagal origin, or have a diagnosis of idiopathic ventricular fibrillation. Some of these patients are diagnosed at follow-up, when the ECG changes spontaneously from normal to the typical pattern of the syndrome. This is also the case for those individuals in whom the disease is unmasked by the administration of an antiarrhythmic drug given for other arrhythmias, for instance atrial fibrillation.

## Diagnosis

The diagnosis of the syndrome is easily obtained by electrocardiography as long as the patient presents the typical ECG pattern and there is a history of aborted sudden death or syncopes caused by a polymorphic VT. The ST segment elevation in V1 to V3 with the right bundle branch block pattern is characteristic. The ST changes are different from the ones observed in acute septal ischemia, pericarditis, ventricular aneurysm and in some normal variants like early repolarization. There are though, ECG's which are not as characteristic, and they are only recognised by a physician who is thinking of the syndrome. There are also many patients with a normal ECG in whom the syndrome can only be recognised a posteriori when the typical pattern appears in a follow-up ECG or after the administration of ajmaline, procainamide or flecainide.

It is possible that the electrocardiographic patterns are different depending on the genetic abnormality. This is the case in other genetic diseases like the long QT syndrome [11]. The mutations that have been discovered give proof of this fact in Brugada syndrome: their ECG's are similar, but not identical. Even though the affected gene is the same, the exact mutation is different. It will be necessary to identify more mutations and make close genotype-phenotype correlation to establish the links. However, we cannot forget the great variability of the ECG in this syndrome, something which will certainly not facilitate analysis.

Additional diagnostic problems are caused by the changes in the ECG induced by the autonomous system and by antiarrhythmic drugs. The study by Myazaki et al [8] was the first one to show the variability of the ECG pattern in the syndrome. Despite the fact that we described the syndrome as a persistent ECG pattern, we soon recognised that it is variable over time, depending on the autonomic interaction and the administration of antiarrhythmic drugs. Adrenergic stimulation decreases the ST segment elevation while vagal stimulation worsens it. The administration of class Ia, Ic and III drugs increase the ST segment elevation. Patients with syncope of unknown cause must be challenged with antiarrhythmic drugs in order to exclude the possibility of this syndrome as a cause of ventricular arrhythmias and syncope.

## Genetic characterisation

In the Brugada syndrome, as in the long QT syndrome, the best candidate genes are those that are responsible for the formation of the cardiac action potential, namely the genes that encode for the cardiac ionic channels. In animal studies, blockade of the calcium-independent 4-aminopyridine-sensitive transient outward potassium current (I_to_) results in surface ECG findings of elevated, downsloping ST-segments due to greater prolongation in the epicardial action potential compared to the endocardium (which lacks a plateau phase). Loss of the action potential plateau (or dome) in the epicardium but not endocardium would be expected to cause ST-segment elevation. Because loss of the dome is caused by an outward shift in the balance of currents active at the end of phase 1 of the action potential (principally I_to_ and I_Ca_), autonomic neurotransmitters like acetylcholine facilitate loss of the action potential dome by suppressing calcium current and augmenting potassium current. b-adrenergic agonists (i.e. isoproterenol, dobutamine) restore the dome by augmenting I_Ca_.  Sodium channel blockers also facilitate loss of the canine right ventricular action potential dome as a result of a negative shift in the voltage at which phase 1 begins.  Hence, I_to_, I_Ca_, and I_Na_ would be good candidate genes to study. Since I_Na_ (SCN5A) has been shown to cause VT/VF in humans (in the long QT syndrome) this gene certainly is worthy of study.

Recently, we reported the findings on six families and several sporadic cases of Brugada syndrome [4].  The families were initially studied by linkage analysis using markers to the known ARVD loci and linkage was excluded.  More recently, seven other families have also excluded linkage to these loci, thus suggesting that the families recruited with the Brugada syndrome to date may indeed by an entity distinct from ARVD. Candidate gene screening using the mutation analysis approach of single strand conformation polymorphism (SSCP) analysis and DNA sequencing was performed and SCN5A was chosen for study.  In three families, mutations in SCN5A were identified  including: 1.- a missense mutation (C-to-T base substitution) causing a substitution of a highly conserved threonine by methionine at codon 1620 (T1620M) in the extracellular loop between transmembrane segments S3 and S4 of domain IV (DIVS3 - DIVS4), an area important for coupling of channel activation to fast inactivation; 2.- a two nucleotide insertion (AA) which disrupts the splice-donor sequence of intron 7 of SCN5A; and 3.- a single nucleotide deletion (A) at codon 1397 which results in an in-frame stop codon that eliminates DIIIS6, DIVS1 - DIVS6, and the carboxy-terminus of SCN5A. Not all the individuals had the typical electrocardiogram at baseline. The diagnosis for genetic purposes was based on the electrocardiographic changes after the administration of ajmaline iv. This test proved 100% sensitive and specific, as all the patients who developed the ST segment elevation had the mutation in the subsequent genetic analysis. Likewise, none of the individuals without the electrocardiographic abnormalities had the genetic abnormality.

Biophysical analysis of the mutants in *Xenopus* oocytes demonstrated a reduction in the number of functional sodium channels in both the splicing mutation and one-nucleotide deletion mutation, which should promote development of reentrant arrhythmias. In the missense mutation, sodium channels inactivated more rapidly than normal. In this case, the presence of both normal and mutant channels in the same tissue would promote heterogeneity of the refractory period, a well-established mechanism of arrhythmogenesis.  Inhibition of the sodium channel I_Na_ current causes heterogeneous loss of the action potential dome in the right ventricular epicardium, leading to a marked dispersion of depolarisation and refractoriness, an ideal substrate for development of reentrant arrhythmias.  Phase 2 reentry produced by the same substrate is believed to provide the premature beat necessary for initiation of the VT and VF responsible for symptoms in these patients.

Mutations in the SCN5A gene were previously shown to be the cause of LQT3, a form of Romano-Ward long QT syndrome. The differences in the clinical findings between LQT3 and Brugada syndrome occur due to the different biophysical results based on the position of the mutations within the gene. Unlike the Brugada syndrome, LQT3 occurs due to an augmentation of late INa carried by SCN5A channels.

## Prognosis and treatment

### Symptomatic patients

Our recent data on 334 patients with the syndrome confirm the generally accepted view that symptomatic patients with this syndrome have an unacceptably high rate of arrhythmic events. Because no effective antiarrhythmic drug or other therapies are available, implantation of a cardioverter-defibrillator is mandatory in these patients. Better understanding of the genetic basis and electrophysiologic mechanisms of the disease may make other therapies possible in the future. Recurrent arrhythmic events were more frequent in patients with aborted sudden death as the presenting symptom as compared to patients with repetitive syncopal episodes. This could suggest a more severe disease in the former group with more frequent and longer-lasting arrhythmias. However, a word of caution is in order because the mean follow-up period of patients with aborted sudden death was significantly longer than the mean follow-up of patients with syncopal episodes. For both categories of symptomatic patients, the recurrence rates approximate a mean of 11% per mean follow-up year (8.8% per year in syncope patients and 13.7% per year in patients resuscitated from sudden cardiac death) and are unacceptably high. The gravity of the problem is amplified when one considers the mean age of the patients.

### Asymptomatic individuals

The major concern at present is with the group of individuals displaying an electrocardiogram compatible with the diagnosis of Brugada syndrome but who are asymptomatic. The initial diagnosis in these individuals was arrived at by different means: In some individuals a spontaneously abnormal electrocardiogram was recorded as part of a routine screening, for instance before surgery. In other individuals, the abnormal electrocardiogram was obtained because of a family history of sudden death. In some, the abnormal electrocardiogram appeared only during treatment with antiarrhythmic drugs given for the treatment of atrial fibrillation or other arrhythmias. Finally, in still others, the abnormal electrocardiogram was obtained only after pharmacologic challenge performed because of the suspicion or documentation of Brugada syndrome in the family. The most recent data allow important conclusions in terms of the management of these asymptomatic individuals.

First, it is confirmed that a *spontaneously abnormal electrocardiogram* is a marker of possible sudden arrhythmic death: 16 of 111 (14%) asymptomatic individuals with a spontaneously abnormal electrocardiogram developed an arrhythmic event during a mean follow-up period of only 27±29 months. The arrhythmic event occurred within one year of diagnosis in 7 individuals, within 2 years in another 3 patients, but after more than 4 years in the remaining 6 individuals. The longest time interval between diagnosis and first arrhythmic event was 10 years. These data demonstrate that a mean follow-up time of slightly more than 2 years underestimates the total number of events that can occur in this population.

Second, the data allowed recognition of groups of asymptomatic individuals with a good prognosis. The group of asymptomatic individuals in whom the *abnormal electrocardiogram was recognized only after pharmacologic challenge* had no events during follow-up. This observation has important implications for the management of individuals who are members of a family with Brugada syndrome. When the individual is asymptomatic and the electrocardiogram is normal, the unmasking of the abnormal electrocardiogram with a drug identifies a carrier of the disease. However, because no events occurred in this group, it is not justified to recommend further investigations in terms of management.

## Conclusions

The syndrome of right bundle branch block, ST segment elevation from V1 to V3 and sudden death is a new entity. This disease is genetically determined and it is different from the long QT syndrome and right ventricular dysplasia. The incidence of sudden death in this syndrome is very high and, at present, sudden death can only be prevented by implanting a cardioverter-defibrillator.
